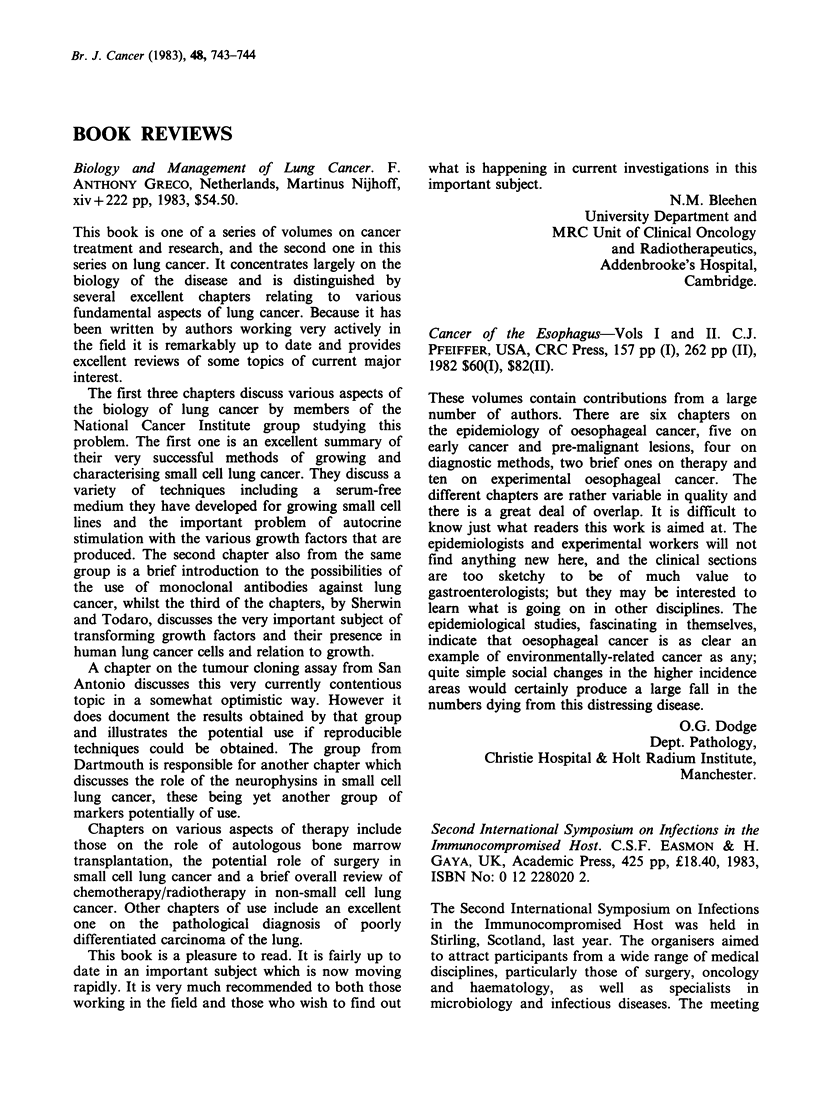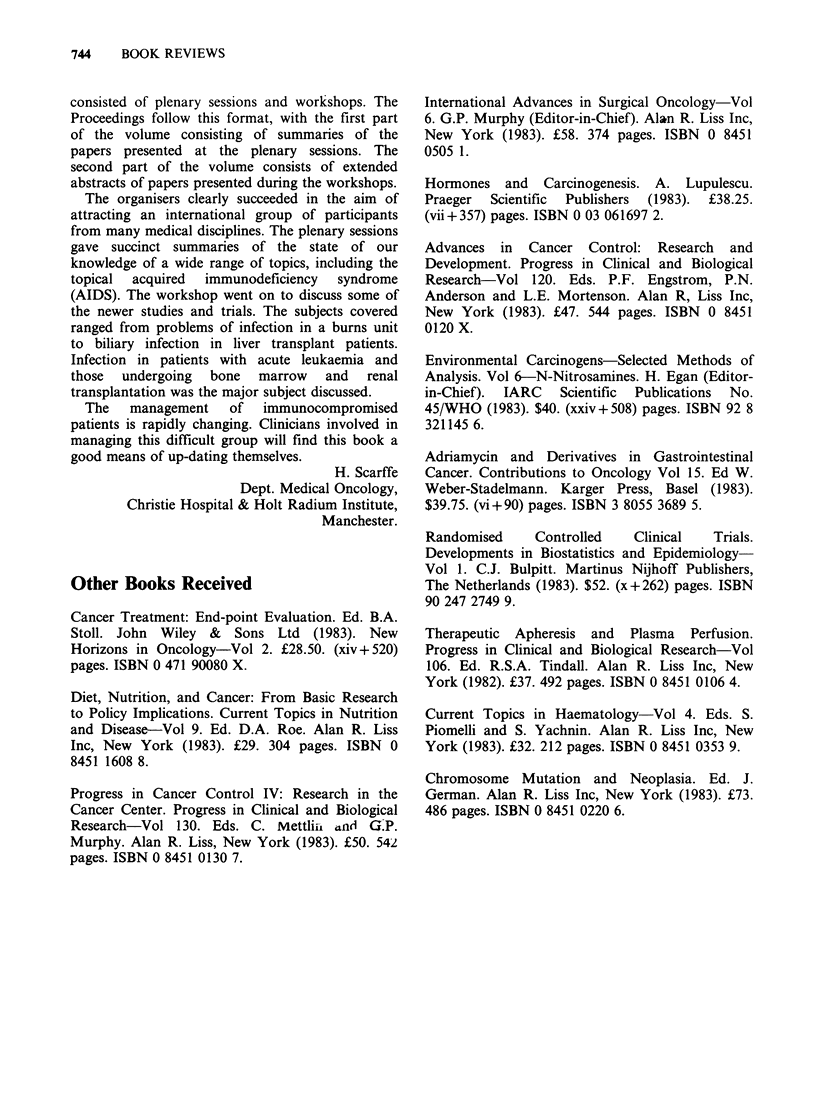# Second International Symposium on Infections in the Immunocompromised Host

**Published:** 1983-11

**Authors:** H. Scarffe


Christie Hospital & Holt Radium Institute,

Manchester.

*Second International Symposium on Infections in the Immunocompromised Host*. C.S.F. EASMON & H. GAYA, UK, Academic Press, 425 pp, £18.40, 1983, ISBN No: 0 12 228020 2.

The Second International Symposium on Infections in the Immunocompromised Host was held in Stirling, Scotland, last year. The organisers aimed to attract participants from a wide range of medical disciplines, particularly those of surgery, oncology and haematology, as well as specialists in microbiology and infectious diseases. The meeting consisted of plenary sessions and workshops. The Proceedings follow this format, with the first part of the volume consisting of summaries of the papers presented at the plenary sessions. The second part of the volume consists of extended abstracts of papers presented during the workshops.

The organisers clearly succeeded in the aim of attracting an international group of participants from many medical disciplines. The plenary sessions gave succinct summaries of the state of our knowledge of a wide range of topics, including the topical acquired immunodeficiency syndrome (AIDS). The workshop went on to discuss some of the newer studies and trials. The subjects covered ranged from problems of infection in a burns unit to biliary infection in liver transplant patients. Infection in patients with acute leukaemia and those undergoing bone marrow and renal transplantation was the major subject discussed.

The management of immunocompromised patients is rapidly changing. Clinicians involved in managing this difficult group will find this book a good means of up-dating themselves.

H. Scarffe
Dept. Medical Oncology,
Christie Hospital & Holt Radium Institute,
Manchester.